# Improving the “urinary side” of acute kidney injury monitoring

**DOI:** 10.1186/s13054-016-1332-3

**Published:** 2016-06-04

**Authors:** Alexandre Toledo Maciel, Daniel Vitorio

**Affiliations:** Imed Research Group, Adult Intensive Care Unit, Hospital São Camilo Pompéia, Av Pompéia 1178, São Paulo, SP 05024-000 Brazil

We read with interest the article by Aniort et al. [1] and would like to make some comments about their findings. Despite the limitations of the study, their data were able to highlight that urine assessment in critically ill patients must not be restricted merely to volume or flow. Recent studies have demonstrated the relevance of assessing both urinary electrolytes [2] as well as the amount of creatinine excreted [3] in order to best monitor acute kidney injury (AKI) development and recovery. There are relevant increments in the information retrieved from urine when its composition is taken into account [4, 5].

In their study, the best renal replacement therapy (RRT) weaning predictor was the amount of excreted urea. This sounds very logical. Physicians are usually aware of the serum consequences of AKI but neglect the urinary phenomena that lead to such consequences. This occurs because we fear the consequences of kidney impairment from the “blood side” because they actually represent the real threats to the patient’s life: uremia, hyperkalemia, acidemia, and hypervolemia. However, it is important to emphasize that consequences are always preceded by causes and, in the case of AKI, the causes can be summarized by the incapacity of the kidneys to excrete properly (urea, potassium, acids, sodium, water, etc.). Therefore, it is intuitive that the early recognition of both AKI development and recovery must focus on urinary excretion rates (the “urinary side”), not on secondary changes in blood.

In their sample, a significant number of patients with theoretically adequate urine output were not able to be weaned from RRT, demonstrating that diuresis improvement is probably a true sign of recovery only when it is followed by an increased capacity to excrete waste products. The prognostic ability of oliguria may vary and could be stratified not only according to flow ranges but also according to urinary urea and creatinine concentration ranges.

Urine biochemistry evaluation seems to be a standard practice in the ICU where the study was developed. We would like to know from the authors whether they think shorter collection periods could replace the 24-h period. If yes, this could make the assessment of excretion rates more feasible and pragmatic. We also would like to ask whether other parameters such as the daily amount of excreted creatinine as well as sodium (total natriuresis) might also have clinically relevant accuracy to predict RRT weaning success.

## Abbreviations

AKI: acute kidney injury
RRT: renal replacement therapy
ICU: Intensive Care Unit

## References

[1] Aniort J, Ait Hssain A, Pereira B, Coupez E, Pioche PA, Leroy C (2016). Daily urinary urea excretion to guide intermittent hemodialysis weaning in critically ill patients. Crit Care.

[2] Maciel AT, Vitorio D. Urine biochemistry assessment in critically ill patients: controversies and future perspectives. J Clin Monit Comput. 2016. doi:10.1007/s10877-016-9871-3. 10.1007/s10877-016-9871-3 27038161

[3] Endre ZH, Pianta TJ, Pickering JW. Timely diagnosis of acute kidney injury using kinetic eGFR and the creatinine excretion to production ratio, E/eG—creatinine can be useful! Nephron. 2016;132(4):312-6. doi:10.1159/000444456. 10.1159/000444456 26950884

[4] Masevicius FD, Vazquez AR, Enrico C, Dubin A (2013). Urinary strong ion difference is a major determinant of plasma chloride concentration changes in postoperative patients. Rev Bras Ter Intensiva.

[5] Moviat M, Terpstra AM, van der Hoeven JG, Pickkers P (2012). Impaired renal function is associated with greater urinary strong ion differences in critically ill patients with metabolic acidosis. J Crit Care.

